# Supplementary material for: Rapid Identification of Nontuberculous Mycobacterium Species from Respiratory Specimens Using Nucleotide MALDI-TOF MS
Source: Microorganisms. 2023 Jul 31;11(8):1975. doi: 10.3390/microorganisms11081975 (PMC10458091; doi:10.3390/microorganisms11081975)
Supplement: Supplementary file 1 [file microorganisms-11-01975-s001.zip › microorganisms-2453445-supplementary.pdf]

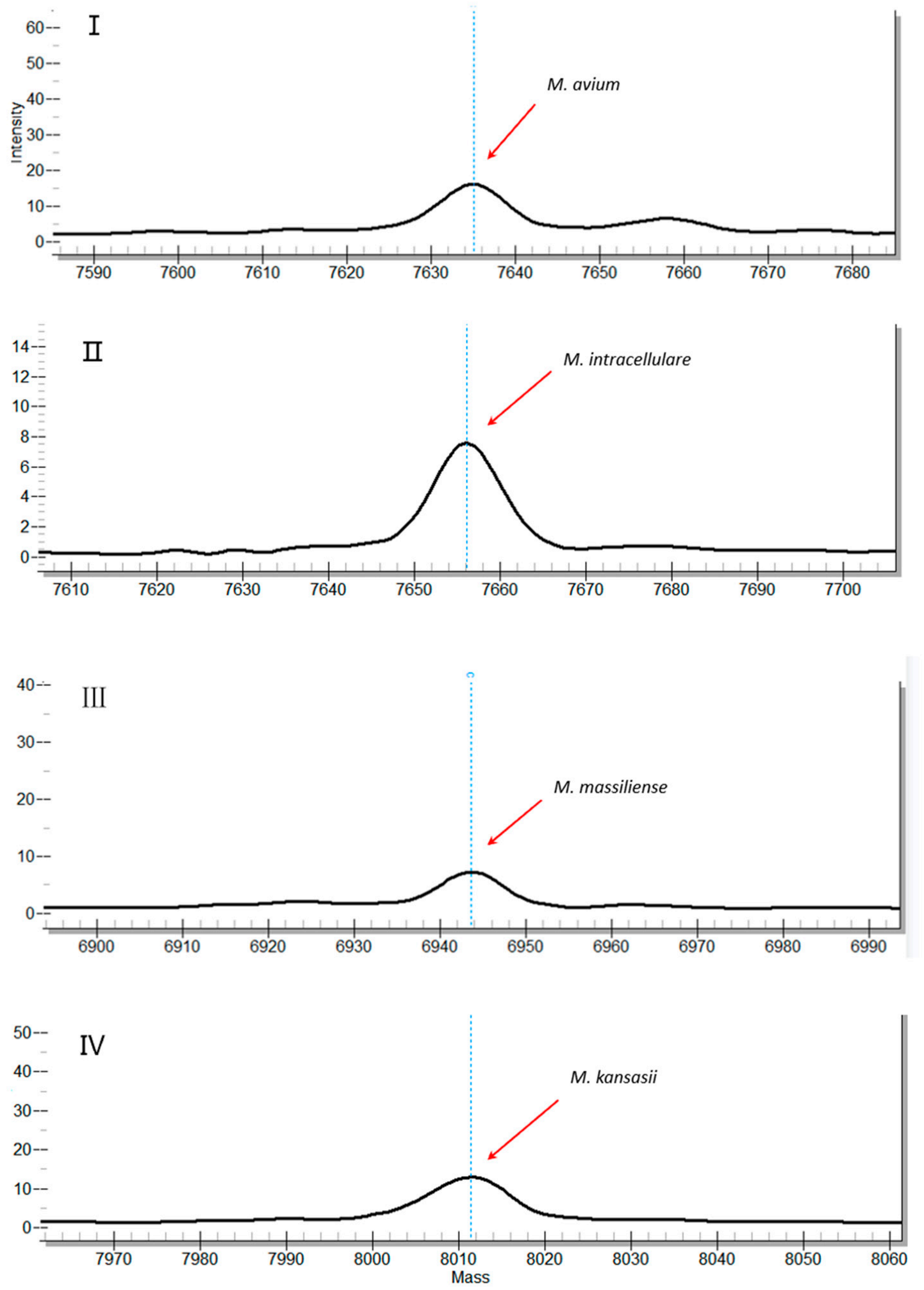

Figure. S1. Nucleotide MALDI-TOF MS mass spectra for representative species. I: *M. avium*, II: *M. intracellulare*, III: *M. massiliense*, IV: *M. kansasii*

Table S1. PCR primers used for identification of nontuberculous mycobacteria.

**Gene locus**	**PCR primer (5'-3')**
T	SEQ ID No. l: ACGTTGGATGCAGTTGATTGCGAGAGCTG
	SEQID No.2: ACGTTGGATGCAGCGCGAACAGCAGTACACCC
RB -1	SEQ ID No.3: ACGTTGGATGATGAACATCGAGACGCT
	SEQ ID No.4: ACGTTGGATGACCGGG TACGACGGGAG
RB -2	SEQ ID No.5: ACGTTGGATGATGAACATCGAGAGTCT
	SEQ ID No.6: ACGTTGGATGCCG TCG ACCGACAG
mce3B-1	SEQ ID No.7: ACGTTGGATGTTCCTGCTGATTGTCTCCGT
	SEQ ID No.8: ACGTTGGATGTCGGGGTTGATGGTCCAGGT
ITS1	SEQID No.9: ACGTTGGATGAATGATTGCCAGACACAGTTCTAG
	SEQ ID No.10:
mce3B-2	SEQ ID No.1l: ACGTTGGATGTGATCCGCTACGACCTCCAG
	SEQ ID No.12: ACGTTGGATGCGATCAGGGCATCATCCAGC
BGAL	SEQ ID No.13: ACGTTGGATGGTTTCGGCGTGAGTAAGGT
	SEQ ID No.14: ACGTTGGATGACGATGTAGATCGGTCGCAC

Table S2. Primers used for PCR extension.

**Gene locus**	**Extension primer (5'-3')**
M. abscessus-bolletii_Thvmidvlate Kinase	SEQ ID No.15: AACGCTGACGAAAAGG
M. massiliense_ Thymidylate Kinase	SEQ ID No.16: CGGGTAAGCGCACTCCCCATTG
M. tuberculosis_mce3B	SEQ ID No.17: CCCAACGTGTCTCTCAAG
M. avium_rpoB	SEQ ID No.18: CCGGGTGGAACATCGACAATGCGO
M. chelonae_-rpoB	SEQID No.19: AACCGGCTGGAACATCGACAAGGGC
M. kansasii_rpoB	SEQ ID No.20: CAGCGGGTGGCAGATCGCTTCGGGA
M.xenipi_-rpoB	SEQ ID No.21: ATGGCTGGAAGATCGCCTAACCO
M. intracellular-chimaera-youngonase_-rpoB	SEQ ID No.22: GGCTGGAACATCGACTCGGGA
M.chimaera_Internal Transcribed Spacer gene-1	SEQ ID No.23: CTGAGACAACACTTCAGGCG
M.intracelluar_Internal Transcribed Spacer gene-1	SEQ ID No.24: GACAACACTGCTGGCA
M. chimaera_Internal Transcribed Spacer gene-1	SEQ ID No.25: TGTGTAATTTCTTTTACTTTAC
M.intracelluare_Internal Transcribed Spacer gene-1	SEQ ID No.26: GAAATGTGTAATTTCTGTTTCTG
M. smegmatis_rpoB	SEQ ID No.27: ATCGACGTCGCCGCTGCGGGG
M. simiae_rpoB	SEQ ID No.28: TCGAACCTGCCGCAGGAGT

M.terrae-A_rpoB	SEQ ID No.29: CCGGCTGGACCGACATA
M. terrae-F_rpoB	SEQ ID No.30: CACATCGATTCTCTGG
M. abscessus_Group_Beta-Galactosidase	SEQ ID No.31: CTATCTGGGGTACTTCGA
M. abscessus_Group_Beta-Galactosidase	SEQ ID No.32: CGCTGGAACATCAAACAGCCTTC
M. abscessus_Group_Beta-Galactosidase	SEQ ID No.32: CGCTGGAACATCAAACAGCCTTC
M.ulceran_rpoB	SEQ ID No.33: CGGCTGGAACATCGACGCCAGCTA
M. mucogenicum_rpoB	SEQ ID No.34: TGGAACATCGACGAGGGG
M. malmoense_rpoB	SEQ ID No.35: ATTTACGTGGCGGGGACCGGT
M.szulgai_rpoB	SEQ ID No.36: CATGGAACATCGATGCAGGT
M. avium_rpoB	SEQ ID No.37: GGGTGGAACATCGACAATGGCC
M.terrae-F_rpoB2	SEQ ID No.38: TCCGGCTGGAACATCGATGCTTTGG
M.peregrinum_rpoB	SEQ ID No.39: AATAAGCCGACAGGGGG
M.immunogenum_rpoB	SEQ ID No.40: CTGGAACATCGACGAGGGT
M.gordonae-A_rpoB	SEQ ID No.41: ATGGAACATCGACGCAGGC
M.gordonae-B_rpoB	SEQ ID No.42: ACGGCTGGAACATCGATGCAGGA
M.fortuitum_rpoB	SEQ ID No.43: AGGCCGGCTGGAACATCGTGGGTACT
M.triplex_rpoB	SEQ ID No.44: TTGAACATCGATTCGGGC
M.branderi_rpoB	SEQ ID No.45: TGGAAGGTCGAAACGGGG
M.intermedium_rpoB	SEQ ID No.46: AGATCGACGTCGCCAATGGCGGT
M.triviale_rpoB	SEQ ID No.47: ATAAGCCGGACACGACCAAC
M.marium_rpoB	SEQ ID No.48: CACGCTGCCCAACCGCGGTGAA

M.lentiflavum_rpoB	SEQ ID No.49: GTGAACGCAGACGGCACGAAGGG
M.scrofulaceum_rpoB	SEQ ID No.50: GCTGGAACATCAACGTTTGG
M.parascrofulaceum_rpoB	SEQ ID No.51: TCCGGCTGGAATATCGATTCGGGA
M.phlei_rpoB	SEQ ID No.52: GCTGGAAGATCGATGAGGGT
M.nonchromogenicum_rpoB	SEQ ID No.53: AATGTTCGACGGTGGGGCGCA
M.arosiense_rpoB	SEQ ID No.54: AACCTGCCGCAGAGGT

Table S3. Procedure used for the iPLEX extension reaction.

Cycles	Cycles	Temperature	Duration
		94 ºC	30 s
40		94 ºC	5 s
	5	52 ºC	5 s
		80 ºC	5 s
		72 ºC	3 min
		4 ºC	Unlimited
